# Role of lncRNA LIPE-AS1 in adipogenesis

**DOI:** 10.1080/21623945.2021.2013415

**Published:** 2021-12-27

**Authors:** Alyssa Thunen, Deirdre La Placa, Zhifang Zhang, John E. Shively

**Affiliations:** a Department of Molecular Imaging and Therapy, Beckman Research Institute of City of Hope, Duarte, CA, USA; b Irell and Manella Graduate School of Biological Sciences, Beckman Research Institute of City of Hope, Duarte, CA, USA

**Keywords:** LIPE, adipogenesis, long non-coding RNA, Ceacam1, apoptosis

## Abstract

Recent studies have identified long non-coding RNAs (lncRNAs) as potential regulators of adipogenesis. In this study, we have characterized a lncRNA, LIPE-AS1, that spans genes *CEACAM1* to *LIPE* in man with conservation of genomic organization and tissue expression between mouse and man. Tissue-specific expression of isoforms of the murine lncRNA were found in liver and adipose tissue, one of which, designated mLas-V3, overlapped the *Lipe* gene encoding hormone-sensitive lipase in both mouse and man suggesting that it may have a functional role in adipose tissue. Knock down of expression of mLas-V3 using anti-sense oligos (ASOs) led to a significant decrease in the differentiation of the OP9 pre-adipocyte cell line through the down regulation of the major adipogenic transcription factors *Pparg* and *Cebpa*. Knock down of mLas-V3 induced apoptosis during the differentiation of OP9 cells as shown by expression of active caspase-3, a change in the localization of LIP/LAP isoforms of C/EBPβ, and expression of the cellular stress induced factors CHOP, p53, PUMA, and NOXA. We conclude that mLas-V3 may play a role in protecting against stress associated with adipogenesis, and its absence leads to apoptosis.

## Introduction

Metabolic disorders, including obesity and type 2 diabetes, are becoming increasingly prevalent in the United States [1,2]. Both of these conditions are associated with alterations to normal adipocyte function. With increased caloric intake, adipose depots undergo hyperplasia, an increase in number, or hypertrophy, and an increase in size, to store the additional triacylglycerides (TAGS) [3,4]. Understanding the generation of new adipocytes is important for understanding their role in disease and identifying potential therapeutic targets.

The differentiation of pre-adipocytes to mature adipocytes is a tightly regulated process involving a network of transcription factors [5–7]. At early stages of adipogenesis, CCAAT/enhancer binding proteins (CEBPs) including C/EBPβ bind to the enhancer and promoter region of the major adipogenic transcription factor Peroxisome Proliferator Activated Receptor gamma (PPARγ) [8–11]. Ectopic expression of PPARγ has been shown to be sufficient to induce adipogenesis in 3T3 cells and PPARγ is required for the differentiation of ES cells to adipocytes [12,13]. C/EBPα is another CCAAT/enhancer binding protein that has also been shown to be a major adipogenic transcription factor. C/EBPα and PPARγ both bind the promoter regions of mature adipocyte genes to drive their expression and lead to full differentiation of adipocytes from their precursors [14–17].

C/EBPβ, a member of the basic leucine zipper (bZip) CCAAT/enhancer binding protein family, plays a large role in early adipogenesis [18–21]. Early studies of its function found that ectopic expression of C/EBPβ in differentiating NIH 3T3 cells was able to drive adipogenesis through the expression of PPARγ [11]. However, the pro-adipogeneic function of C/EBPβ has mostly been attributed to the longer isoforms of C/EBPβ, the two Liver Activating Proteins, LAP and LAP* [19,22,23]. The shorter isoform, Liver Inhibitory Protein, LIP, is anti-adipogenic and its overexpression in pre-adipocytes blocks differentiation [19]. Thus, the ratio of LAP/LIP plays a role in adipogenesis and other differentiation pathways including osteoclastogenesis, monocyte differentiation, and differentiation of haematopoietic stem cells in mouse [24–26]. LAP and LIP are also targets for proteasome degradation that are responsible for changes in their expression patterns [23,27].

C/EBP Homologous Protein CHOP and mTORC1 have been found to be involved in the regulation of the LAP/LIP ratio [28]. mTORC1 regulates the translation of the LIP isoform [28,29], and CHOP has been found to stabilize the LIP isoform [29]. A number of studies have shown that early in adipogenesis, CHOP can form a heterodimer with C/EBPβ and inhibit adipogenesis [30–32]. In later stages of differentiation, expression of CHOP decreases, releasing C/EBPβ and allowing differentiation to continue [30,31].

CHOP is also important in ER stress-induced apoptosis where it acts downstream of stress signals and can regulate the apoptotic pathway [29,33,34]. The process of adipogenesis involves ER stress since the cells need to highly express a number of lipid storage and lipolysis proteins in mature versus immature adipocytes [35,36]. However, if the cell is unable to resolve the ER stress, apoptosis is triggered.

Mature adipocytes store TAGs that are hydrolysed to free fatty acids by the action of adipocyte resident lipases, providing the organism with a major energy source [37,38]. Among the three steps of fatty acid cleavage from TAGs, hormone-sensitive lipase encoded by *LIPE*, plays a critical role in that its activation is regulated by both catecholamines and insulin [37,38].

During the course of our studies on *CEACAM1*, a gene highly expressed in liver where lipogenesis occurs, we became interested in its potential role in adipocytes, since *Ceacam1* knock out mice develop visceral obesity with age, especially in male mice [39,40]. Although *Ceacam1* is not expressed in adipocytes, we noticed that a long non-coding RNA designated as 4732471J01RiK in mice and LIPE-AS1 in humans, spanned the genes *Ceacam1* to *Lipe* [41]. This, observation prompted us to examine potential functions of this lncRNA in the liver and adipose tissue.

Non-coding RNAs have more recently been found to play a role in various cellular pathways including adipogenesis and lipolysis [42–47]. Long non-coding RNAs (lncRNAs) are non-coding RNAs that are longer than 200 nucleotides. They have been found to act as miRNA sponges, and among other functions, act as scaffolds for chromatin modifiers that can activate or repress gene expression [48]. The chromatin organization of LIPE-AS1 is highly conserved from mouse to man suggesting a shared function and regulation of its expression. Since LIPE-AS1 is expressed as a number of splice isoforms (http://genome.ucsc.edu), tissue-specific expression is also possible. As a first step to explore these possible roles, we characterized one of the murine splice forms that partially overlapped *Lipe* and studied its tissue expression and potential role in adipogenesis in a model system.

To characterize the function of the murine lncRNA, we utilized the bone stroma derived pre-adipocyte cell-line model OP9 [49], since their differentiation to mature adipocytes is not passage number sensitive like the pre-adipocyte cell-line 3T3-L1 [49–51]. OP9 cells treated with dexamethasone, IBMX, and insulin are able to differentiate within five days making them a suitable model for the transient knock down of the third variant of the murine *Lipe* antisense lncRNA (mLas-V3), the subject of this study [49].

## Results

### Conservation of gene organization and expression of LIPE anti-sense lncRNAs

**Figure 1.**
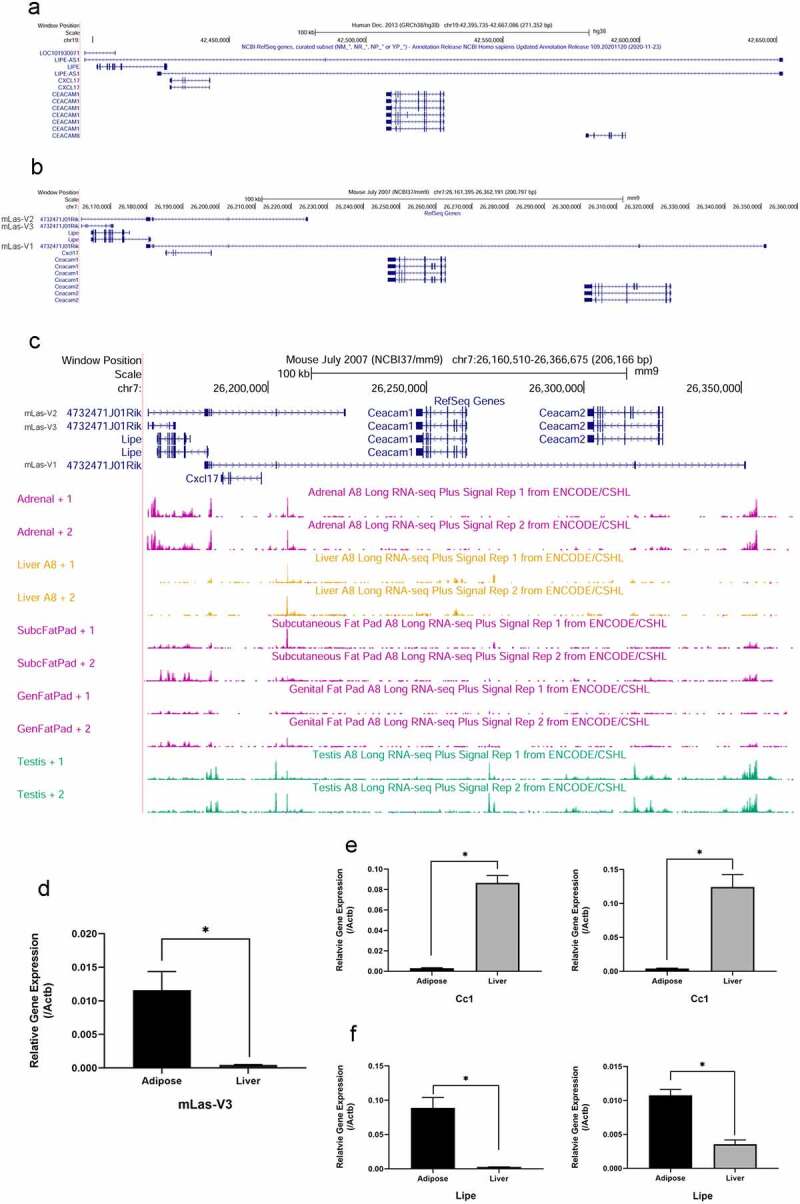
**Genomic organization of human *LIPE-AS1* and murine *mLas* genes and expression of *mLas-V3* in murine adipose and liver**. There is conserved organization and expression of LIPE-AS1 and mLas in mouse and man as shown in the UCSC genome Browser (http://genome.ucsc.edu). (a) UCSC Genome Browser tracks of the human genome (GRCh38/hg38) showing the organization of the LIPE-AS1 variants and their anti-sense genes: *LIPE, CXCL17, CEACAM1*, and *CEACAM8*. (b) UCSC Genome Browser tracks of the mouse genome (NCBI/mm9) showing the organization of the three 4732471J01RIK variants (mLas-V1, mLas-V2, and mLas-V3) and their anti-sense genes: *Lipe, Cxcl17, Ceacam1*, and *Ceacam2*. (c) UCSC Genome Browser tracks of the mouse genome showing the tissue Long RNA-seq Plus Signal data from the Encode/CSHL data set in mouse tissues where LIPE-AS1 is also shown to be highly expressed in humans. Liver data is included although LIPE-AS1 and mLas expression are low in this tissue. (d) qRT-PCR data of *mLas-V3* expression, (e) *Ceacam1* expression, and (f) *Lipe* expression in epididymal adipose tissue compared to liver. For all tissues, qRT-PCR, n = 5 mice. Data normalized to beta-actin using 2^−ΔCt^ method and analysed using the Student’s t-test.

*LIPE-AS1* is a long non-coding RNA (lncRNA) encoded on the plus strand of chromosome 19 in man and is anti-sense to the gene that encodes hormone-sensitive lipase (HSL), *LIPE. LIPE* is expressed in adipose tissue and HSL is involved in the breakdown of diglycerides to monoglycerides. This lncRNA and its variants are anti-sense to additional genes including *CXCL17, CEACAM1*, and *CEACAM8* (Figure 1a). This genomic organization is also conserved in mouse on chromosome 7. In mice, there are three splice variants of the lncRNA identified by RIKEN and annotated as 4732471J01RiK [52]. These lncRNAs are also anti-sense to *Lipe, Cxcl17, Ceacam1*, and *Ceacam2* (Figure 1b). It should be noted that *Ceacam2*, an apparent gene duplication event of *Ceacam1*, is unique to mouse [53]. This conservation of genomic organization suggests that this lncRNA may have an important functional role and may be regulated similarly to or involved in the regulation of the genes to which it is anti-sense.

In addition to conserved genomic organization, there is also conserved tissue expression between mouse and man. In humans, HPA RNA-seq data [54] and GTEx data [55] have found high expression of *LIPE-AS1* in subcutaneous and visceral adipose tissue, adrenal glands, breast-mammary tissue, and testis, where *LIPE* is also expressed. Low expression was found in the liver which is a tissue that expresses high levels of CEACAM1 [41]. This tissue expression pattern is similar in mice. However, it is unclear if there might also be tissue-specific expression of the splice variants. Long-RNA sequencing data from the ENCODE/Cold Spring Harbour Lab suggests that there may be differential expression patterns [56,57]. The shortest variant, which we have termed murine *Lipe* anti-sense variant 3 (mLas-V3), only overlaps the *Lipe* gene, and based on the UCSC Genome Browser tracks that show long RNA-seq plus signals in selected tissues based on high expression, expression of mLas-V3 is present in the adrenal gland, subcutaneous and visceral adipose tissue, and testis. There is low to little signal in the liver (Figure 1c). This difference in expression between adipose and liver tissue was confirmed by qRT-PCR (Figure 1d). However, the UCSC Genome Browser long-RNA sequencing signal tracks for other tissues show RNA-seq signals for the two other variants (Figure 1c). In the liver, in the variant region that overlaps *Ceacam1*, the tracks with RNA-seq signals suggest that this longer splice variant of mLas, mLas-V1, is expressed in the liver (Figure 1c). Since *Ceacam1* is more highly expressed in the liver (Figure 1e) while *Lipe* is more highly expressed in adipose tissue (Figure 1f), it is possible that this longer variant, mLas-V1, could be expressed in the liver. Given that the expression and genomic organization of this lncRNA is conserved between mouse and man, we decided to investigate its function in adipose tissue.

### mLas-V3 expression is regulated through adipogenesis

**Figure 2.**
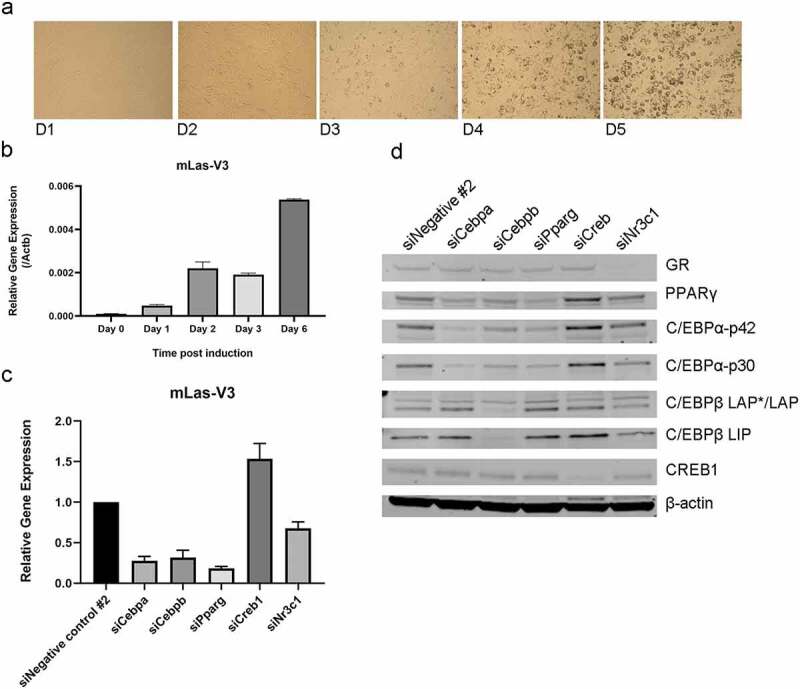
**Expression of mLas-V3 during adipogenesis**. OP9 cells were used as a pre-adipocyte cell model. (a) Images of OP9 cells taken over the 5 days of differentiation. (b) qRT-PCR analysis of *mLas-V3* over time course normalized to beta-actin (n = 4). (c) qRT-PCR of *mLas-V3* expression two days post start of differentiation in which siRNA knock down of indicated genes was performed at day −1. Data was normalized to beta-actin and compared to siNegative control using the 2^−ΔΔCt^ method, n = 3 independent experiments. (d) Representative Western blot data confirming knock down of protein expression.

To characterize and study the function of mLas-V3, the bone stroma derived pre-adipocyte cell-line model OP9 (Figure 2a) was used. qRT-PCR analysis of mLas-V3 expression through differentiation with dexamethasone, IBMX, and insulin showed that expression of mLas-V3 increases as the cells differentiate into mature adipocytes (Figure 2b). This pattern of expression suggests that mLas-V3 might be regulated by adipogenic transcription factors. Using the JASPAR transcription factor-binding profile database [58], the promoter region was found to include potential binding sites for C/EBP, PPARγ, CREB1, and the glucocorticoid receptor, all transcription factors associated with adipogenesis (Supplementary Table 1) [7]. siRNAs (Supplementary Table 2) were used to test if these transcription factors might be involved in the regulation of this lncRNA. After transfection with siRNAs to these five transcription factors, OP9 cells were differentiated as above and then collected at day two to examine the effect of the siRNA mediated knockdowns. Knockdown of these transcription factors, with the exception of CREB1, led to a decrease in the expression of mLas-V3 (Figure 2c). However, analysis of expression of these proteins revealed increases in PPARγ and C/EBPα of about 30%, after CREB1 knock down, a result that could explain the increase in expression of mLas-V3 (Figure 2d). These results demonstrate that mLas-V3 expression is regulated by these adipogenic transcription factors.

### Subcellular localization and characterization of mLas-V3

**Figure 3.**
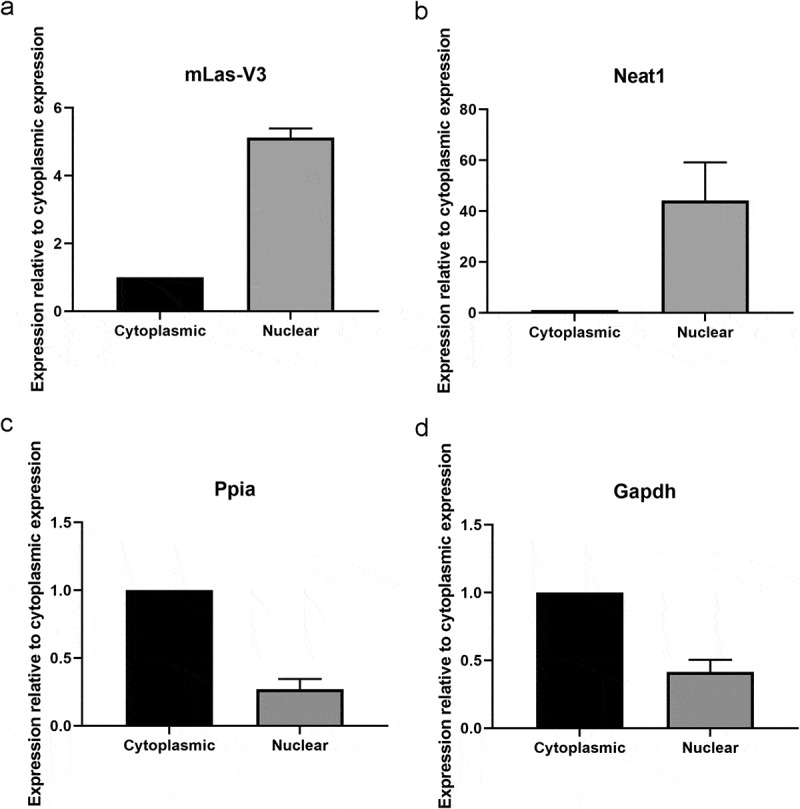
**Nuclear localization of mLas-V3**. OP9 cells were fractionated at day 5 of differentiation into nuclear and cytoplasmic RNA fractions and analysed by qRT-PCR. Expression of (a) *mLas-V3*, (b) nuclear lncRNA *Neat1*, (c) *Ppia* and (d) *Gapdh* cytoplasmic markers. Data normalized to Gapdh using the 2^−ΔCt^ method, n = 3.

Using RNA from differentiated OP9 cells, mLas-V3 was found to be polyadenylated using 3ʹ Rapid Amplification of cDNA Ends (RACE) (Supplementary Figure S1). The 5ʹ end of the lncRNA was also confirmed using 5ʹ RACE (Supplementary Figure S1). Polyadenylation suggests that the lncRNA can be transported from the nucleus to the cytoplasm. However, nuclear and cytoplasmic RNA fractionation followed by qRT-PCR showed that mLas-V3 is a nuclear enriched lncRNA (Figure 3a) similar to the nuclear lncRNA control Neat1 (Figure 3b) [59], while the cytoplasmic controls *Ppia* and *Gapdh* wer localized mainly to the cytoplasm (Figure 3c) [60]. The nuclear localization of mLas-V3 suggested that locked nucleic acid GapmeR anti-sense oligos (ASOs), rather than siRNAs, would be necessary to target nuclear lncRNAs since they utilize RNAse H cleavage to degrade both nuclear and cytoplasmic RNA targets [61,62].

### Knockdown of mLas-V3 leads to decrease in mature adipocytes

**Figure 4.**
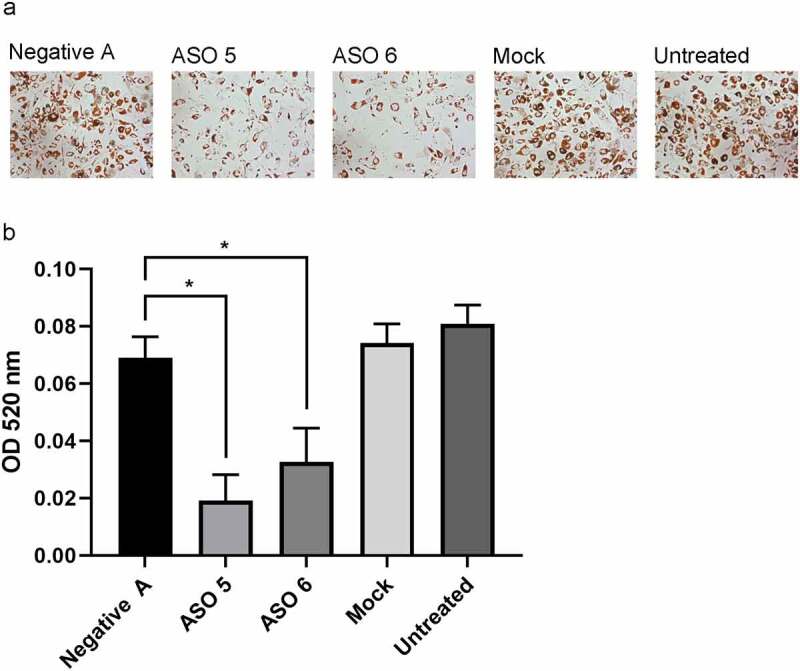
**Knock down of mLas-V3 blocks adipogenesis**. OP9 cells were transfected with control or targeting ASOs or mock transfected, subjected to differentiation, and at day 5 were fixed and stained with Oil Red O. (a) Representative images were taken at 10x magnification. (b) Oil Red O staining was quantified by measuring absorbance of solution extracted Oil Red O from day 5 differentiated cells. n = 3 wells.

To determine if knockdown of mLas-V3 would affect differentiation of OP9 pre-adipocytes, OP9 cells were mock transfected or transfected with a scrambled antisense oligo (ASO) negative control or mLas-V3 targeted ASOs (ASO 5 and ASO 6). Two targeted ASOs were used to confirm the effect of the knockdown. The cells were then differentiated over a five-day period and lipid droplet accumulation was assessed using Oil Red O staining. At day five, there was a decrease in mature adipocytes, as determined by lipid droplet staining, in ASO 5 and 6 transfected cells compared to the controls (Figure 4a, b). A majority of the control cells showed accumulation of lipid droplets showing successful differentiation and that only a small portion of the cells were still proliferating.

### Knockdown of mLas-V3 leads to a decrease in expression of adipogenic transcription factors

**Figure 5.**
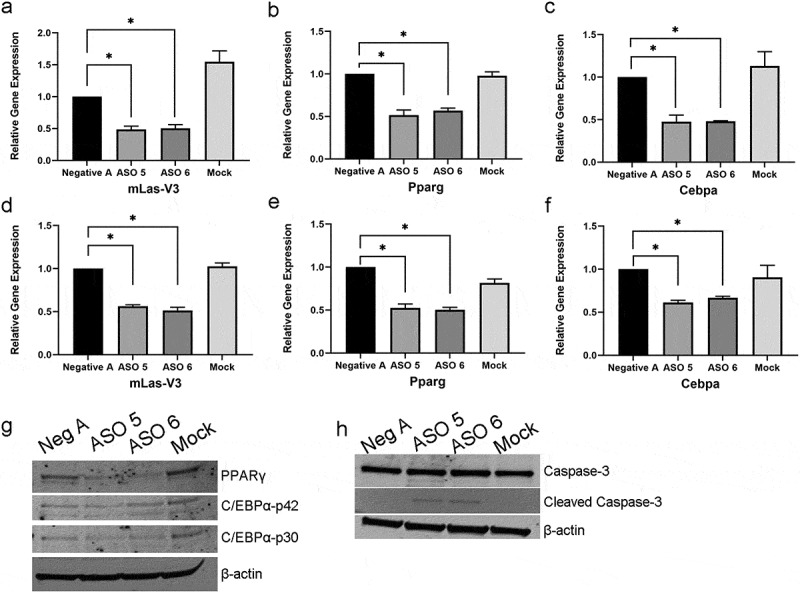
**Expression levels of *mLas-V3, Pparg* and *Cebpa* in *mLas-V3* knock down cells at day 2 and day 1 of differentiation**. OP9 cells were transfected with control or targeting ASOs or mock transfected. RNA was isolated for qRT-PCR analysis after one and two days post induction of differentiation. (a) Expression of mLas-V3 confirmed knock down at day 2 of differentiation. qRT-PCR of (b) *Pparg* and (c) *Cebpa* at day 2. qRT-PCR of levels of expression of (d) *mLas-V3*, (e) *Pparg*, and (f) *Cebpa* in cells treated with ASOs or mock transfected and collected one day after induction of differentiation. Data normalized using beta-actin and compared to Negative A control using the 2^−ΔΔCt^ method. (g) Protein expression of PPARγ, C/EBPα, and β-actin in day 1 differentiated cells using total cell lysate. (h) Protein expression of total Caspase-3, cleaved Caspase-3, and β-actin in day 1 differentiated cells using cytoplasmic cell lysates.

To determine if the decrease in mature adipocytes after mLas-V3 knockdown was due to changes in adipogenic transcription factor gene expression, day two samples were collected and analysed by qRT-PCR. At day two, in addition to a significant decrease in mLas-V3 expression in ASO treated cells (Figure 5a), there was about a 40% decrease in *Pparg* and *Cebpa* expression compared to the negative ASO scrambled control (Figure 5b, c).

The down regulation of these adipogenic transcription factors was then investigated at an earlier stage of differentiation. Cells transfected with control or ASOs 5 and 6 collected 1 day after differentiation were analysed by qRT-PCR and immunoblot analysis to determine the expression of these transcription factors. With the knock down of mLas-V3 (Figure 5d), there was a significant down regulation of *Pparg* and *Ceba* expression compared to the negative ASO scrambled control (Figure 5e, f). Immunoblots using total protein lysates also showed a down regulation of *Pparγ* and *Cebpα* expression in ASO 5 and 6 treated cells compared to controls (Figure 5g).

**Figure 6.**
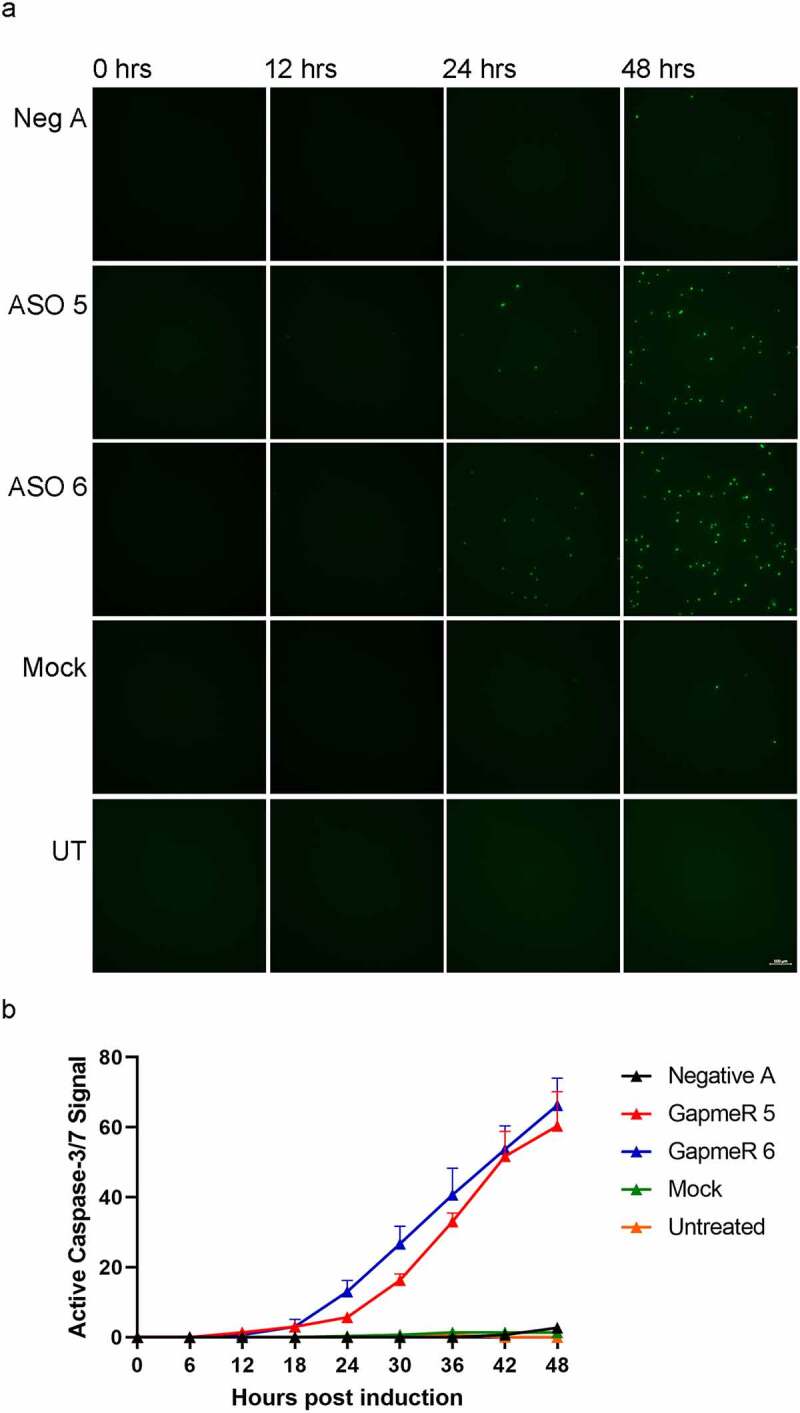
**Knock down of mLas-V3 in differentiating adipocytes and apoptosis**. (a) Time course imaging of transfected cells using CellEvent ReadyProbe Caspase-3/7 reporter. The green signal represents active Caspase-3/7. UT, untreated. (b) Active caspase-3/7 was quantified over the time course. Each dot was counted and data was plotted as ±SEM.

Utilizing 5ʹ FAM labelled ASOs, the targeted ASOs were found to accumulate in the nucleus at day one, then in apoptotic bodies starting from day 2 (Supplementary Figure S2). It has been reported that apoptotic bodies can include DNA and RNA [63,64]. Caspase-3 cleavage was detected in the cytoplasm of day 1 ASO targeted cells further demonstrating that the cells are undergoing apoptosis (Figure 5h). Experiments testing the addition of a pan-caspase inhibitor to cells that have been treated with ASOs to determine if blocking caspase activity would rescue adipogenesis were attempted but were not successful (data not shown). To further investigate apoptosis as an outcome of mLas-V3 knock down by ASOs, OP9 cells were transfected with ASOs or mock transfected and imaged over a two day time period post induction of differentiation using a non-fluorescent reagent that has a peptide recognized by caspase 3 and 7 attached to a DNA binding dye. When the peptide is cleaved by active caspase 3 or 7, the dye is able to bind DNA and fluoresce. Representative images are shown in Figure 6a and quantified in Figure 6b. Starting at 18 hours after the induction of differentiation, active caspase 3/7 was detected in ASO treated cells while there was none detected in the control treated cells. Interestingly, there was a slight delay with the effect of ASO 5 compared to ASO 6 suggesting that these two ASOs might have slightly different time courses of action.

### Nuclear accumulation of the C/ebpβ LIP isoform and expression of stress related proteins

**Figure 7.**
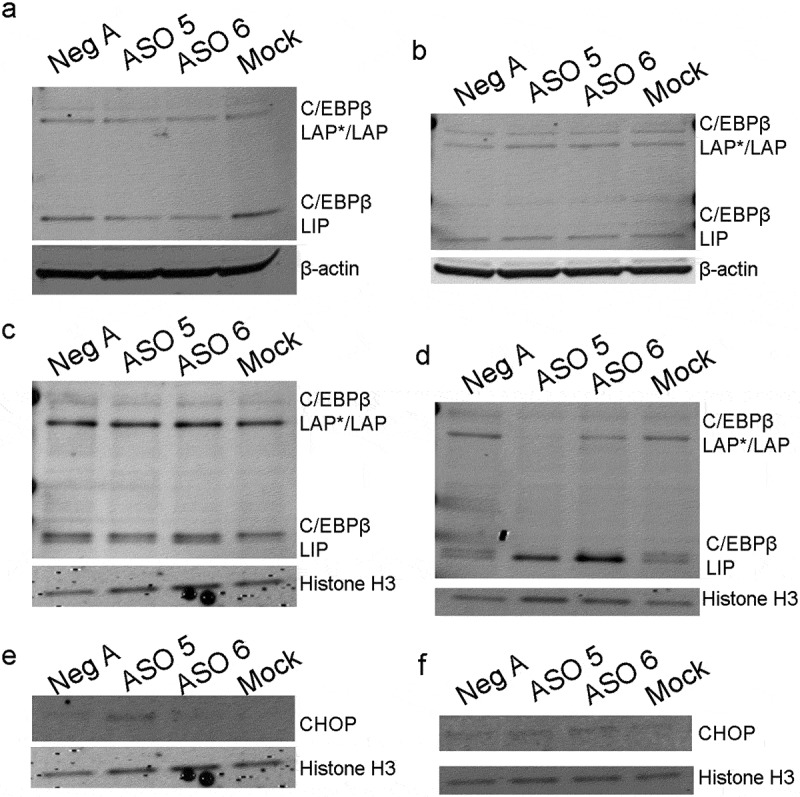
**Protein expression of C/EBβ isoforms and CHOP in cells transfected with mLas-V3 ASOs undergoing differentiation**. (a) One day and (b) two days post differentiation. Nuclear protein expression of C/EBPβ and Histone H3 in cells transfected with ASOs and collected (c) one day or (d) two days post differentiation. (e) Nuclear expression of CHOP in cells transfected with ASOs and collected one day post differentiation. (f) Nuclear expression of CHOP in cells transfected with ASOs and collected two days post differentiation.

Due to the decrease in gene expression of both *Pparg* and *Cebpa*, it was important to examine the expression of the upstream transcription factor *Cebpb*. However, after analysis of protein expression from total protein lysates at both day one and day two after differentiation, there were no apparent changes in the expression of C/EBPβ due to mLas-V3 knock down (Figure 7a,b). Nonetheless, since mLas-V3 is a nuclear enriched lncRNA, its role may be specific to the nuclear compartment. Therefore, nuclear protein lysates were collected from ASO and mock transfected cells at one and two days after differentiation. There was no change in C/EBPβ expression in ASO treated cells compared to controls at day one post differentiation (Figure 7c); however, at day two post differentiation, there was about a 50% decrease in the expression of the larger C/EBPβ isoforms LAP* and LAP in the nuclear fractions. There also was an increase in the accumulation of the smaller isoform, LIP, in this fraction (Figure 7d), suggesting that changes in the localization of the C/EBPβ isoforms could be responsible for the phenotype.

C/EBPβ-LIP, a negative regulator of adipogenesis, is able to bind to DNA but is missing an activation domain [19,22,65]. It has also been found to have a potential role in apoptosis due to ER stress through its interaction with the transcription factor CHOP [27,29]. For example, Chiribau et al. [29] found that in early ER stress LIP is degraded in the proteasome but in late ER stress it associates with CHOP and translocates to the nucleus. CHOP has been associated with the down regulation of anti-apoptotic genes leading to apoptosis [29,33,34,66]. CHOP was expressed in the nucleus of ASO 5 treated cells one day after differentiation (Figure 7e). However, there was little to no expression of CHOP in ASO 6 treated cells at the same time point. By day two after differentiation, there was expression of CHOP in both ASO 5 and ASO 6 treated cells (Figure 7f).

**Figure 8.**
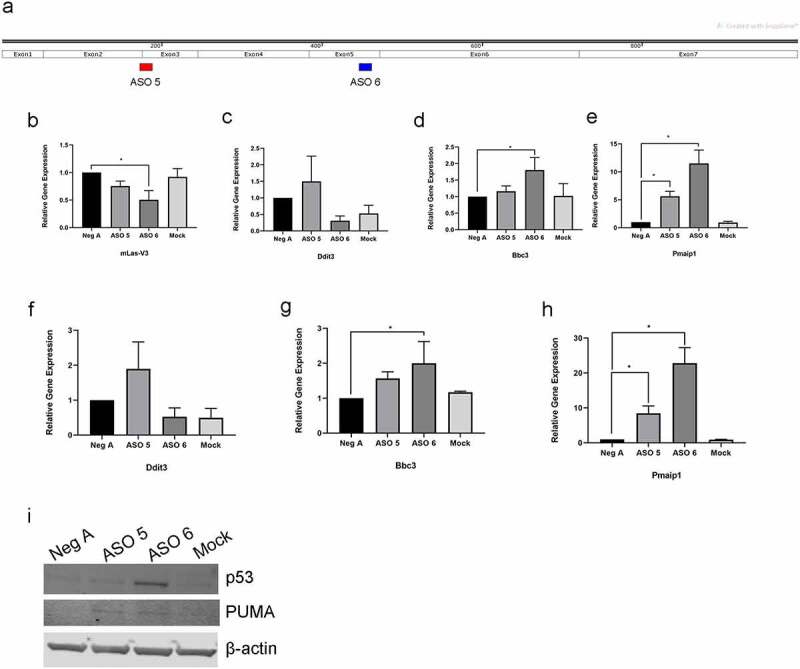
**Knockdown of mLas-V3 by ASO 5 and 6 lead to activation of different stress pathways**. (a) Exon structure of mLas-V3 showing regions targeted by ASO 5 and ASO 6. Image generated using SnapGene software. qRT-PCR levels of expression of (b) *mLas-V3*, (c) *Ddit3*, (d) *Bbc3*, and (e) *Pmaip1* in cells treated with ASOs or mock transfected and collected 6 hours post induction of differentiation. qRT-PCR levels of expression of (f) *Ddit3*, (g) *Bbc3*, and (h) *Pmaip1* in cells treated with ASOs or mock transfected and collected one day post induction of differentiation. Data normalized using beta-actin and compared to Negative A control using the 2^−ΔΔCt^ method. (i)Protein expression of p53, PUMA, and β-actin in day 1 differentiated cells using total cell lysate.

The expression of CHOP after ASO knockdown of mLas-V3 suggests that loss of mLas-V3 expression could lead to cellular stress and the activation of the intrinsic apoptosis pathway. Importantly, ASO 5 and ASO 6 target two different regions of mLas-V3 (Figure 8a) and we have noted that they have slightly different time courses of action (Figure 6b). Further investigations of factors involved in stress and the intrinsic apoptosis pathway showed that 6 hours post induction of differentiation, there was a decrease in the expression of mLas-V3 in ASO 5 and ASO 6 treated cells (Figure 8b). At 6 hours post induction of differentiation, there was an increase in the gene expression of *Ddit3* (which encodes CHOP) in ASO 5 treated cells compared to the control but not in ASO 6 treated cells (Figure 8c). However, in ASO 6 treated cells, there was a significant increase in the expression of *Bbc3* (which encodes PUMA) in ASO 6 treated cells compared to the negative control but no increase in expression in ASO 5 treated cells (Figure 8d). Both ASO 5 and ASO 6 treated cells lead to increased expression of *Pmaip1* (which encodes NOXA) 6 hours post induction of differentiation (Figure 8e). These expression patterns are similar at one day post differentiation, as ASO 5 treated cells showing an increase in *Ddit3* expression (Figure 8f) and ASO 6 treated cells also showed a significant increase in *Bbc3* expression compared to controls (Figure 8g). Both ASO 5 and ASO 6 treatments also lead to increased expression of *Pmaip1* at one day post differentiation (Figure 8h).

PUMA and NOXA are pro-apoptotic BH3-only proteins [67–70] that can be transcriptionally upregulated by p53 when cells are under stress [69,70]. p53 is upregulated due to various cellular stressors including genotoxic stress as well as ER stress [67,68,71,72]. We found an increase in expression of p53 in ASO 6 treated cells 1-day post differentiation (Figure 8i). Notably, PUMA is expressed in both ASO 5 and ASO 6 treated cells 1-day post differentiation (Figure 8i). Therefore, these results suggest that different regions of mLas-V3 may contribute to different patterns of gene expression; however, knocking down expression of mLas-V3 at either region leads to apoptosis due to cellular stress.

## Discussion

In this study, we have characterized a splice isoform of the murine lncRNA *Lipe*-antisense gene we have designated as mLas-V3. mLas-V3 is conserved at the genomic level in both mouse and man. We chose to focus on the mLas-V3 mouse variant of the lncRNA because it overlaps with the *Lipe* gene responsible for the expression of hormone-sensitive lipase in adipocytes. Since mLas-V3 also showed high expression in adipose tissue, we hypothesized that it might also play a role in adipogenesis. Regarding its genomic conservation between mouse and man, it has been suggested that conserved lncRNAs need not have sequence conservation [73,74]. Instead, there may be structural, functional, or genomic organization conservation of lncRNAs [73]. Since this lncRNA shows conservation of both genomic organization and tissue expression patterns in both species, we hypothesized a functional role for mLas-V3 in adipose tissue.

Many recent studies have found roles for lncRNAs in adipose tissue, especially in the process of adipogenesis. A study by Sun et al. identified multiple lncRNAs that when knocked down would lead to a block of adipogenesis [43]. Other groups have identified the functions of specific lncRNAs in adipogenesis. Examples include the lncRNA ADINR, which was determined by Xiao et al. to be involved in the expression of C/EBPα through the recruitment of chromatin modifiers [75]. Another lncRNA, MIR31HG, was found by Huang et al. to play a role in histone modification leading to expression of FABP4 and adipocyte differentiation of human stem cells [76].

We found that similar to some of the previously described lncRNAs, knockdown of mLas-V3 leads to decreased adipogenesis. Knockdown of mLas-V3 leads not only to decreased expression of the major adipogenenic transcription factors PPARγ and C/EBPα, but also to altered localization of C/EBPβ isoforms with increased nuclear accumulation of the negative regulator isoform, LIP, and expression of cell stress-related proteins and apoptosis, including CHOP, p53, and PUMA. Interestingly, it appears that the different regions of the lncRNA may target different pathways since the two ASOs used to knock down mLas-V3 led to activation of apoptosis through two different cellular stress pathways. ASO 5 led to apoptosis likely through an ER stress pathway through the increased expression of nuclear CHOP while ASO 6 knockdown led to expression of p53 which could have been induced by ER or genotoxic stress.

Li et al. showed that the LAP/LIP ratio also changes throughout the induction of ER stress in C6 glioma cells [27]. They showed that in late stage ER stress there was an increase in LIP levels and this was partially due to increased stability of this isoform. They also showed that C/EBPβ might have a role in CHOP expression during ER stress. In a follow-up study by Chiribau et al. it was shown that in early ER stress, LIP was found in the cytoplasm where it was degraded by the proteasome [29]. In later ER stress, LIP associated with CHOP which protected it from degradation. In addition, Chiribau et al. found that LIP can help CHOP to translocate to the nucleus where CHOP can regulate genes involved in apoptosis [29].

The role and function of noncoding RNAs in modulation of ER stress and apoptosis has been an area of increased study. Many microRNAs have been found to be involved in the regulation of proteins involved in the unfolded protein response which is a response to ER stress [77]. One identified function of lncRNAs is acting as a sponge for miRNAs [48]. In a study by Geng et al., the authors found that the lncRNA GAS5 could lead to apoptosis by sponging miR-21 which then led to the increased expression of the miR-21 target TSP-1, a pro-apoptotic protein [78]. lncRNA genes can encode miRNA genes that can also be regulated by, and in turn, regulate ER stress. A study by Kato et al. found that lnc-MGC contains a megacluster of microRNAs and that the expression of this lncRNA was regulated by CHOP [79]. The authors also found that the targets of the microRNAs included genes involved in ER stress [79].

In addition to functioning as sponges, lncRNAs have also been found to play a role in apoptosis through more direct mechanisms. PTEN has pro-apoptotic activity and it was found that the PTENpg1 anti-sense RNA isoform α could act as a negative regulator by recruiting epigenetic modifiers to the PTEN promoter leading to a decrease in transcription [80,81]. Wu et al. also found that the lncRNA GOLGA2P10 was regulated by CHOP and that it had a protective effect against ER stress-induced apoptosis by increasing the expression of anti-apoptotic BCL-xL protein potentially through a post-transcriptional mechanism [82]. These studies suggest that the pathways controlling the unfolded protein response, ER stress, and apoptosis may have an additional layer of regulation by non-coding RNAs.

In our study, we found that the knock down of mLas-V3 leads to apoptosis as shown by the expression of cleaved caspase-3 (Figure 5e). Cells treated with antisense oligos expressed cell stress-related proteins. We also showed nuclear accumulation of the C/EBP β-LIP isoform that has been previously been found to play a role in ER stress through its interaction with CHOP. Cells treated with ASO 5 prior to differentiation had nuclear expression of the ER stress-related protein CHOP (Figure 7e). Cells treated with ASO 6 showed an increased expression of p53 and increased gene expression of pro-apoptotsis markers *Bbc3* and *Pmaip1*. Thus, our findings suggest that mLas-V3 may play a role in regulating stress and suppressing apoptosis.

Further studies are required to better understand the upstream mechanism of this non-coding lncRNA and its role in apoptosis. This includes the identification of factors that directly interact with mLas-V3, studies that are difficult due to the relative low abundance of this lncRNA. While overexpression studies of mLas-V3 may also help to better define the role of this lncRNA, they may suffer from non-physiological off-target effects. Downstream studies that further define C/EBPβ isoform expression changes may also be revealing. Finally, examination of the mechanism of the generation of the mature mLas-V3 splice form may reveal other aspects of the regulation of adipogenesis. That type of study requires additional definition of exon-intron boundaries of the parent gene that spans over 8,900 nucleotides.

In conclusion, we have characterized splice variant mLas-V3, a lncRNA conserved in mouse and man at both the genomic and tissue expression level in adipose tissue. This conservation suggests that this lncRNA may have an important role in function since its knockdown in pre-adipogenic OP9 cells blocked adipogenesis due to decreased expression of major adipogeneic transcription factors and induction of apoptosis. We present evidence that apoptosis in these cells is triggered by a stress response leading to expression of p53, PUMA, and CHOP.

## Materials and methods

### UCSC genome browser data analysis

Genome browser images from the Mouse July 2007 (NCBI37/mm9) genome and the Human December 2013 (GRCh38/hg38) genome (http://genome.ucsc.edu). Mouse genome images show the NCBI RefSeq track and human genome images show NCBI RefSeq Curated track updated annotation release 109.20201120 [83,84]. ENCODE/CSH long RNA-seq track (ENCODE Transcriptome group data) is also shown with data from the GEO accession numbers: GSM900188 (adults 8 weeks adrenal), GSM900191 (adults 8 week subcutaneous fat pad), GSM900190 (adults 8 weeks genital fat pad), GSM900193 (adult 8 week testis), GSM900195 (adult 8 weeks liver) produced by the Gingeras Cold Spring Harbour Laboratories group and the Center for Genomic Regulation (Barcelona) [56,57].

### Animal studies

All mice used were of the C56BL/6 background in accordance with IACUC protocol 11,033 approved by the City of Hope Institutional Animal Care and Use Committee, in accordance with the National Institute of Health Office of Laboratory Animal Welfare guidelines. Wild type C57BL/6 mice were purchased from Jackson Laboratory.

### Cell culture

OP9 Cells (CRL-2749) were purchased from American Type Culture Collection (ATCC). Cells were cultured in MEM-α media without nucleosides (Gibco 12,561) with 20% foetal bovine serum. Differentiation was induced similar to the method described by Hasbargen et al [85]. Cells were differentiated by adding a media containing MEM-α media without nucleosides, 10% foetal bovine serum, 0.5 mM IBMX (Sigma I7018) and 1 μM dexamethasone (Sigma D8893). 48 hours after inducing differentiation, media was changed to MEM-α media without nucleosides, 10% foetal bovine serum with 1 μg/mL of insulin (Sigma I0516).

### Transfection studies

For ASO transfections, cells were seeded to be confluent at the time of transfection. Cells were forward transfected with Lipofectamine 3000 (Invitrogen L3000001) following manufacturer’s instructions using a final concentration of 50 nM Qiagen GapmeR ASOs. Catalogue numbers are listed in Supplementary Table 2. Five hours post transfection, media was changed to differentiation media.

For siRNA transfections, cells were seeded at a density of 10,000 cells/cm^2^ the day before transfection. Cells were then forward transfected using Silencer Select siRNA purchased from Ambion Life Technologies at a final concentration of 20 nM. Catalogue numbers are listed in Supplementary Table 2. Media was changed to differentiation media 24 hours post transfection. Cells were collected for RNA and protein analysis on day two of differentiation.

### RLM-3ʹ RACE

RNA was isolated from day 6 differentiated OP9 cells and reverse transcribed according to manufacturer’s instructions (Ambion RLM RACE Kit). Two rounds of PCR were used to isolate a specific product. Primer sequences are listed in Supplementary Table 2. The PCR product was cloned using the TOPO TA cloning kit (Thermo Fisher 450,071) and sequenced. Sequences analysed using SnapGene software from Insightful Science; available at snapgene.com.

### TSO 5ʹ RACE

RNA was isolated from day 6 differentiated OP9 cells and prepared according to the New Englad BioLabs 5ʹ RACE protocol using a Template Switching Oligo (TSO) [86]. Briefly, an Oligo d(T) primer (NEB S1327S) was annealed to the RNA template. Template Switching RT Enzyme mix (NEB M0466S) was then used for reverse transfection. PCR using primers specific to the TSO oligo and mLas-V3 were used to amplify the 5ʹ region. Primer sequences are listed in Supplementary Table 2. The PCR product was cloned using the Zero Blunt TOPO PCR kit (Thermo Fisher 450,159) and sequenced.

### Nuclear and cytoplasmic fractionation

Nuclear and cytoplasmic fractions were isolated from OP9 cells with the NE-PER Nuclear and Cytoplasmic Extraction Reagents (Thermo Scientific 78,833). For RNA, TRIzol was added to the cytoplasmic fraction and the nuclear pellet and processed as described in the following section. For protein, nuclear and cytoplasmic fractions were isolated following manufacturer’s instructions.

### RNA isolation, cDNA synthesis, and qRT-PCR

For tissue RNA, epididymal adipose and livers were collected from five age matched mice. Liver tissue was first ground in liquid nitrogen and 100 mg of homogenized tissue was collected into a Precellys homogenizer tube (Bertin Instruments). Adipose tissue (50 to 100 mg) was homogenized in a Precellys homogenizer tube. One mL of TRIzol reagent (Invitrogen 15,596,026) was added to samples and homogenized using a Precellys homogenizer. The samples were then spun and the supernatant was transferred to a new tube. RNA was then isolated following manufacturer’s instructions. Purified RNA was then re-precipitated using 5 M ammonium acetate (Invitrogen AM9070G) per manufacturer’s instructions. Equal amounts of RNA were then used to synthesize cDNA using the Maxima First Stand cDNA Synthesis Kit with dsDNase (Thermo Scientific K1672).

Cellular RNA was isolated using TRIzol reagent (Invitrogen 15,596,026) and the Direct-zol RNA isolation kit (Zymo Research) following manufacturer’s instructions, including the on column DNase I digestion step. After elution, cDNA was synthesized from equal amounts of RNA using the Maxima cDNA synthesis kit. An amount equivalent to 20 ng of original RNA was used in each qRT-PCR reaction. Pre-designed primer probes were ordered from Integrated DNA Technologies (IDT). Catalogue numbers are listed in Supplementary Table 2. TaqMan Fast Advanced Master Mix (Applied Biosystems 4,444,557) was used for qRT-PCR on the Bio-Rad CFX 96 instrument. Data were normalized to either Actb or Gapdh and analysed using either 2^−ΔCt^ or 2^−ΔΔCt^ method as noted in the figure legends. Either a Student’s t-test or One-way Anova was used for statistical analysis (Graphpad 9.0.2). Results were considered significant (*) when p < 0.05.

### Immunoblot analysis

Total cellular protein was collected using RIPA buffer (Thermo Fisher Scientific 89,900) supplemented with Halt Protease and Phosphatase Inhibitor Cocktail, EDTA-free (Thermo Fisher Scientific 78,441). Cells were lysed on ice for 30 minutes. Lysates were then spun at 16,000xg for 10 minutes and the supernatant were collected into a new tube. Protein concentrations were measured using the Pierce BCA assay kit (Thermo Fisher Scientific 23,225).

Equal amounts of protein were separated by SDS-PAGE and transferred onto PVDF membranes using the iBlot system (Invitrogen IB401001). Blots were blocked for 30 minutes at room temperature using the Intercept TBS blocking buffer (LI-COR 927–60,001). Primary antibodies were diluted in Intercept T20 TBS antibody diluent buffer (LI-COR 927–65,001) at 1:1000 [PPARγ (Cell Signalling Technology C26H12), C/EBPα (CST D56F10), C/EBPβ(GeneTex 1H7), CREB (CST 86B10), Caspase-3 (CST D3R6Y), Cleaved Caspase-3 (CST 5A1E), p53(1C12)] or 1:500 [CHOP (CST D46F1) PUMA (CST E1S7A)] and incubated overnight at 4°C. Primary control antibodies [β-actin (CST D6A8) and Histone H3 (CST D1H2)] were diluted 1:2500 and incubated one hour at room temperature. Blots were washed with TBST and then probed with either anti-mouse or anti-rabbit LI-COR IRDye 680 or 800 secondary antibodies diluted 1:20,000 in Intercept antibody diluent for one hour at room temperature. Blots were imaged using the LI-COR Odyssey CLX system.

### Active Caspase-3/7 image analysis

CellEvent Caspase-3/7 Green ReadyProbes reagent (R37111) from Thermo Fisher was used following manufacturer’s instruction. Cells were plated and transfected in a 24-well plate and induced to differentiate as described above. Three positions per well were imaged every 15 minutes on a Zeiss Axio Observer Z1 live cell microscope at 10X over 48 hours. Images were analysed using ZEN 3.1 software. Active caspase-3/7 signal was quantified every 6 hours for each position and the average was plotted ±SEM.

### Lipid staining, Imaging, and quantification

Cells seeded in a 24-well plate format and transfected with ASOs were induced to differentiate as described above. On day 5, cells were washed with PBS and then fixed with 4% formaldehyde before being stained with Oil Red O. Images were acquired on a Zeiss Axio Observer Z1 microscope at 10x magnification.

For quantification of Oil Red O, cells were seeded and transfected as described above. On day 5, the Adiopgenesis Assay Kit from Millipore (ECM950) was used to quantify Oil Red O stain following manufacturer’s instruction.

## Supplementary Material

Supplemental Material

## Data Availability

Raw data available upon request of senior author.
