# Supplementary material for: Role of lncRNA LIPE-AS1 in adipogenesis
Source: Adipocyte. 2021 Dec 27;11(1):11–27. doi: 10.1080/21623945.2021.2013415 (PMC8726699; doi:10.1080/21623945.2021.2013415)
Supplement: Supplemental Material [file KADI_A_2013415_SM4074.zip › supplementary/Supplemental Data_revised.docx]

**Role of lncRNA LIPE-AS1 in adipogenesis**

Alyssa Thunen^1,2^, Deidre La Placa^1^, Zhifang Zhang^1^, and John E. Shively^1^*

^1^Department of Molecular Imaging and Therapy and ^2^Irell and Manella Graduate School of Biological Sciences, Beckman Research Institute of City of Hope, 1550 East Duarte Road, Duarte, CA 91010

*Corresponding author: John E. Shively

Email: [jshively@coh.org](mailto:jshively@coh.org)

Running title: LIPE-AS1 and lipogenesis

**Keywords:** LIPE, adipogenesis, long non-coding RNA, CEACAM1, apoptosis

**Supplemental data.**

**Supplemental S1. JASPAR predicted binding sites.**

JASPAR transcription factor binding site predictions for the region 2kb upstream of mLas-V3 exon 1. Downloaded from <http://jaspar.genereg.net/>.

| Matrix ID | Name | Score | Relative score | Sequence ID | Start | End | Strand | Predicted sequence |
| --- | --- | --- | --- | --- | --- | --- | --- | --- |
| MF0002.1 | bZIP CREB/G-box-like subclass | 11.0736 | 1 | Sequence | 1928 | 1933 | - | TGACGT |
| MA0018.2 | CREB1 | 9.92666 | 0.939871 | Sequence | 1926 | 1933 | - | TGACGTCT |
| MA0065.2 | Pparg::Rxra | 14.5779 | 0.908113 | Sequence | 216 | 230 | + | CAGGGTGAAAGGGCA |
| MA0102.2 | CEBPA | 8.53514 | 0.90321 | Sequence | 1288 | 1296 | + | TGGCACAAT |
| MA0102.1 | Cebpa | 9.34845 | 0.89888 | Sequence | 1288 | 1299 | + | TGGCACAATCCC |
| MF0002.1 | bZIP CREB/G-box-like subclass | 7.27931 | 0.880796 | Sequence | 1597 | 1602 | - | TGATGT |
| MA0113.2 | NR3C1 | 10.5379 | 0.879732 | Sequence | 1618 | 1632 | + | AACACAGTGAGTTCA |
| MA0018.2 | CREB1 | 8.00121 | 0.869358 | Sequence | 1926 | 1933 | + | AGACGTCA |
| MA0113.2 | NR3C1 | 9.67892 | 0.869038 | Sequence | 1633 | 1647 | - | TGCACAGACTGGCCT |
| MA0018.2 | CREB1 | 7.93742 | 0.867022 | Sequence | 1370 | 1377 | - | GGACGCCA |
| MA0102.2 | CEBPA | 7.50936 | 0.866259 | Sequence | 905 | 913 | - | TTTTACAAG |
| MA0065.2 | Pparg::Rxra | 11.737 | 0.86185 | Sequence | 783 | 797 | - | GCGGAGGAAGGGGCA |
| MF0006.1 | bZIP cEBP-like subclass | 7.317 | 0.859436 | Sequence | 1287 | 1295 | + | ATGGCACAA |
| MF0002.1 | bZIP CREB/G-box-like subclass | 6.47298 | 0.855464 | Sequence | 1252 | 1257 | - | TGAGGT |
| MF0002.1 | bZIP CREB/G-box-like subclass | 6.47298 | 0.855464 | Sequence | 1659 | 1664 | + | TGAGGT |
| MF0002.1 | bZIP CREB/G-box-like subclass | 6.47298 | 0.855464 | Sequence | 1773 | 1778 | - | TGAGGT |
| MA0113.2 | NR3C1 | 8.48469 | 0.854171 | Sequence | 339 | 353 | - | GGAACGGTAAGAACT |
| MA0102.2 | CEBPA | 7.16209 | 0.85375 | Sequence | 606 | 614 | + | TGGCCCAAC |
| MA0102.1 | Cebpa | 7.75508 | 0.849563 | Sequence | 606 | 617 | + | TGGCCCAACACC |
| MF0006.1 | bZIP cEBP-like subclass | 6.91814 | 0.847974 | Sequence | 1288 | 1296 | - | ATTGTGCCA |
| MA0102.2 | CEBPA | 6.84124 | 0.842192 | Sequence | 711 | 719 | + | TCTAGCAAT |
| MA0102.2 | CEBPA | 6.78017 | 0.839992 | Sequence | 906 | 914 | + | TTGTAAAAT |
| MA0102.1 | Cebpa | 7.4061 | 0.838762 | Sequence | 624 | 635 | - | TTGTTCAAGCCT |
| MA0102.2 | CEBPA | 6.73727 | 0.838447 | Sequence | 1544 | 1552 | - | ATTCCCAAC |
| MF0006.1 | bZIP cEBP-like subclass | 6.4468 | 0.834428 | Sequence | 989 | 997 | + | ATTATGTGA |
| MA0102.2 | CEBPA | 6.59278 | 0.833242 | Sequence | 627 | 635 | - | TTGTTCAAG |
| MF0002.1 | bZIP CREB/G-box-like subclass | 5.71601 | 0.831682 | Sequence | 1201 | 1206 | + | TGACAT |
| MF0002.1 | bZIP CREB/G-box-like subclass | 5.71601 | 0.831682 | Sequence | 1370 | 1375 | + | TGGCGT |
| MF0002.1 | bZIP CREB/G-box-like subclass | 5.71601 | 0.831682 | Sequence | 1855 | 1860 | + | TGACAT |
| MF0002.1 | bZIP CREB/G-box-like subclass | 5.71601 | 0.831682 | Sequence | 1926 | 1931 | + | AGACGT |
| MF0006.1 | bZIP cEBP-like subclass | 6.33815 | 0.831306 | Sequence | 1641 | 1649 | - | GTTGCACAG |
| MA0113.2 | NR3C1 | 6.62232 | 0.830986 | Sequence | 888 | 902 | + | GGATCTCAATTTTCT |
| MA0113.2 | NR3C1 | 6.56559 | 0.83028 | Sequence | 1618 | 1632 | - | TGAACTCACTGTGTT |
| MA0065.2 | Pparg::Rxra | 9.77142 | 0.829843 | Sequence | 749 | 763 | - | CTAAGGGAGGGGTCT |
| MA0113.2 | NR3C1 | 6.52038 | 0.829717 | Sequence | 1351 | 1365 | + | AAATCAGAGTGACTC |
| MA0102.2 | CEBPA | 6.47187 | 0.828886 | Sequence | 379 | 387 | - | TGTCGCAAA |
| MF0006.1 | bZIP cEBP-like subclass | 6.1171 | 0.824954 | Sequence | 56 | 64 | + | CTTGCATCT |
| MA0102.1 | Cebpa | 6.92562 | 0.823891 | Sequence | 628 | 639 | + | TTGAACAAGGCT |
| MA0102.2 | CEBPA | 6.33264 | 0.823871 | Sequence | 628 | 636 | + | TTGAACAAG |
| MF0006.1 | bZIP cEBP-like subclass | 5.99484 | 0.82144 | Sequence | 718 | 726 | + | ATTGCTTAC |
| MA0102.1 | Cebpa | 6.7525 | 0.818532 | Sequence | 1541 | 1552 | - | ATTCCCAACAGC |
| MF0006.1 | bZIP cEBP-like subclass | 5.88139 | 0.81818 | Sequence | 992 | 1000 | - | ATTTCACAT |
| MA0113.1 | NR3C1 | 9.43637 | 0.818107 | Sequence | 451 | 468 | - | GGGTAGATCATGCCCCAG |
| MA0102.1 | Cebpa | 6.72525 | 0.817689 | Sequence | 1284 | 1295 | - | TTGTGCCATGCC |
| MF0006.1 | bZIP cEBP-like subclass | 5.8527 | 0.817356 | Sequence | 905 | 913 | + | CTTGTAAAA |
| MF0006.1 | bZIP cEBP-like subclass | 5.83341 | 0.816801 | Sequence | 906 | 914 | - | ATTTTACAA |
| MA0102.2 | CEBPA | 6.13227 | 0.816653 | Sequence | 1287 | 1295 | - | TTGTGCCAT |
| MA0102.1 | Cebpa | 6.65936 | 0.81565 | Sequence | 376 | 387 | - | TGTCGCAAATTC |
| MA0018.2 | CREB1 | 6.52875 | 0.815434 | Sequence | 253 | 260 | - | TGCCGCCA |
| MA0113.1 | NR3C1 | 9.28696 | 0.815181 | Sequence | 1238 | 1255 | - | AGGTGGAGCCTGTACTAG |
| MA0113.2 | NR3C1 | 5.2752 | 0.814216 | Sequence | 1208 | 1222 | + | CAGACTAAGAGTTCC |
| MF0006.1 | bZIP cEBP-like subclass | 5.71818 | 0.81349 | Sequence | 858 | 866 | + | CTTACATCT |
| MA0018.2 | CREB1 | 6.46894 | 0.813244 | Sequence | 1305 | 1312 | - | TGAGGCTA |
| MA0102.1 | Cebpa | 6.57507 | 0.813041 | Sequence | 906 | 917 | + | TTGTAAAATGGG |
| MA0102.1 | Cebpa | 6.51745 | 0.811258 | Sequence | 992 | 1003 | + | ATGTGAAATGCT |
| MA0113.1 | NR3C1 | 9.06981 | 0.810928 | Sequence | 337 | 354 | - | GGGAACGGTAAGAACTTT |
| MA0102.2 | CEBPA | 5.92447 | 0.809168 | Sequence | 992 | 1000 | + | ATGTGAAAT |
| MA0113.2 | NR3C1 | 4.83661 | 0.808756 | Sequence | 1156 | 1170 | - | GGGACAGAGCTTCCT |
| MA0113.2 | NR3C1 | 4.77645 | 0.808007 | Sequence | 106 | 120 | - | TGAAAGAGGTGTTCT |
| MA0113.2 | NR3C1 | 4.562 | 0.805337 | Sequence | 1156 | 1170 | + | AGGAAGCTCTGTCCC |
| MF0006.1 | bZIP cEBP-like subclass | 5.42747 | 0.805136 | Sequence | 934 | 942 | + | ATGGCAACA |
| MA0113.2 | NR3C1 | 4.5226 | 0.804847 | Sequence | 1240 | 1254 | - | GGTGGAGCCTGTACT |
| MA0113.2 | NR3C1 | 4.32065 | 0.802333 | Sequence | 769 | 783 | - | AGGAAAAGATGGACT |
| MA0102.2 | CEBPA | 5.73177 | 0.802226 | Sequence | 1641 | 1649 | + | CTGTGCAAC |
| MA0113.2 | NR3C1 | 4.23586 | 0.801277 | Sequence | 1616 | 1630 | + | ATAACACAGTGAGTT |
| MF0006.1 | bZIP cEBP-like subclass | 5.2778 | 0.800835 | Sequence | 562 | 570 | + | ATGGCATAG |
| MA0113.2 | NR3C1 | 4.18047 | 0.800588 | Sequence | 1042 | 1056 | - | AATAATAACAGTTCT |

**Supplemental Table S2**. **Oligonucleotides used in study**.

| **siRNAs** | **Ambion** | **Sequence** |
| --- | --- | --- |
| Silencer Select Negative control #2 | Cat # 4390846 |  |
| Silencer Select Cebpa | s63854 | Sense: 5’-AAAGCUGAGUUGUGAGUUAtt-3’  Antisense: 5’-UAACUCACAACUCAGCUUUct-3’ |
| Silencer Select Cebpb | s201157 | Sense: 5’-AGUAAUCACUUAAAGAUGUtt-3’  Antisense: 5’-ACAUCUUUAAGUGAUUACUca-3’ |
| Silencer Select Pparg | s211378 | Sense: 5’-GCAUCUCCACCUUAUUAUUtt-3’  Antisense: 5’-AAUAAUAAGGUGGAGAUGCag-3’ |
| Silencer Select Creb 1 | s232196 | Sense: 5’-GCAGUGCUUGAAAACCAAAtt-3’  Antisense: 5’-UUUGGUUUUCAAGCACUGCca-3’ |
| Silencer Select Nr3c1 | s67066 | Sense: 5’-GUAUAUGGGAGAGACCGAAtt-3’  Antisense: 5’-UUCGGUCUCUCCCAUAUACag-3’ |
|  |  |  |
| **LNA Gapmers** | **Qiagen** |  |
| Negative control A | 339515LG00000002 | 5’-AACACGTCTATACGC-3’ |
| ASO 5 | 339523LG00201398 | 5’-CAAAGGTGCTGGGCTA-3’ |
| ASO 6 | 339523LG00201399 | 5’-AGGCTCACAGTTACCA-3’ |
|  |  |  |
| **Primer probes** | **IDT** |  |
| mLas-V3 | Mm.PT.58.16547709.g | 5’-ATCCCTTCACAGTTCACACC-3’  5’-CTCTGGGTCTATGGCGAATC-3’ |
| Pparg | Mm.PT.58.31161924 | 5’-TGCAGGTTCTACTTTGATCGC-3’  5’-CTGCTCCACACTATGAAGACAT-3’ |
| Cebpa | Mm.PT.58.30061639.g | 5’-TCATTGTCACTGGTCAACTCC-3’  5’-ACAAGAACAGCAACGAGTACC-3’ |
| Actb | Mm.PT.39a.22214843.g | 5’- GACTCATCGTACTCCTGCTTG3’  5’- GATTACTGCTCTGGCTCCTAG-3’ |
| Lipe | Mm.PT.58.30708147 | 5’- CTCGTTGCGTTTGTAGTGC-3’  5’- CTGCAAGAGTATGTCACGCTA-3’ |
| Lipe | Mm.PT.58.6342082 | 5’- CCATATTGTCTTCTGCGAGTGT-3’  5’- GGCGAAAAGGCAAGATCAAAG-3’ |
| Ceacam1 | Mm.PT.58.41165586 | 5’- GTTGTCAGAAGGAGCCAGAT-3’  5’- CATAATATTTGACCCAACACAAGGAG-3’ |
| Ceacam1 | Mm.PT.58.10295193 | 5’- GGTTGCTGGGAATTGAAGTTC-3’  5’- AGGAAGTCTGGCGGATCT-3’ |
| Neat1 | Mm.PT.56a.32296870.g | 5’ –GCCCAGTCTCTTTTATTTCCCA-3’  5’- GCTTTGCCACTGAATACATCC-3’ |
| Ppia | Mm.PT.39a.2.gs | 5’-TTCACCTTCCCAAAGACCAC-3’  5’-CAAACACAAACGGTTCCCAG-3’ |
| Gapdh | Mm.PT.39a.1 | 5’- GTGGAGTCATACTGGAACATGTAG-3’  5’- AATGGTGAAGGTCGGTGTG-3’ |
| Pmaip1 | Mm.PT.58.31607669 | 5’- GCACACTCGTCCTTCAAGT-3’  5’- CCGGACATAACTGTGGTTCT-3’ |
| Bbc3 | Mm.PT.58.7446951 | 5’- AGAGATTGTACATGACCCTCCA-3’  5’- GACCTCAACGCGCAGTA-3’ |
| Ddit3 | Mm.PT.58.30882054 | 5’- GACTCAGCTGCCATGACTG-3’  5’- GCGACAGAGCCAGAATAACAG-3’ |
|  |  |  |
| **Race primers** | **IDT** |  |
| 3' Outer Gene Specific Primer |  | 5’-CTCTGGGTCTATGGCGAATC-3’ |
| 3' Inner Gene Specific Primer |  | 5’-TCACCCAACTACCCAGGACAG-3’ |
| 5' Gene Specific Primer |  | 5’-TCCAGAGTCATGGGAGGTTG-3’ |

**Supplemental Figure S1**. **RACE analysis of mLas-V3 using RNA from differentiated OP9 cells**. (**a**) Confirmation of polyadenylation and 3’ sequence of mLas-V3 by 3’ RACE. Sequence alignment image shows the junction of 3’ end of the lncRNA the and 3’ RACE adaptor sequence. Image generated using SnapGene software. Gel image of 3’ outer and inner RACE PCR products. (**b**) Confirmation of 5’ end using TSO 5’ RACE. 5’ RACE sequence alignment showing junction of TSO sequence and UCSC genome browser annotated exon 1 of mLas-V3. Gel image showing 5’ RACE product. Lower band on gel image found to be non-specific product.


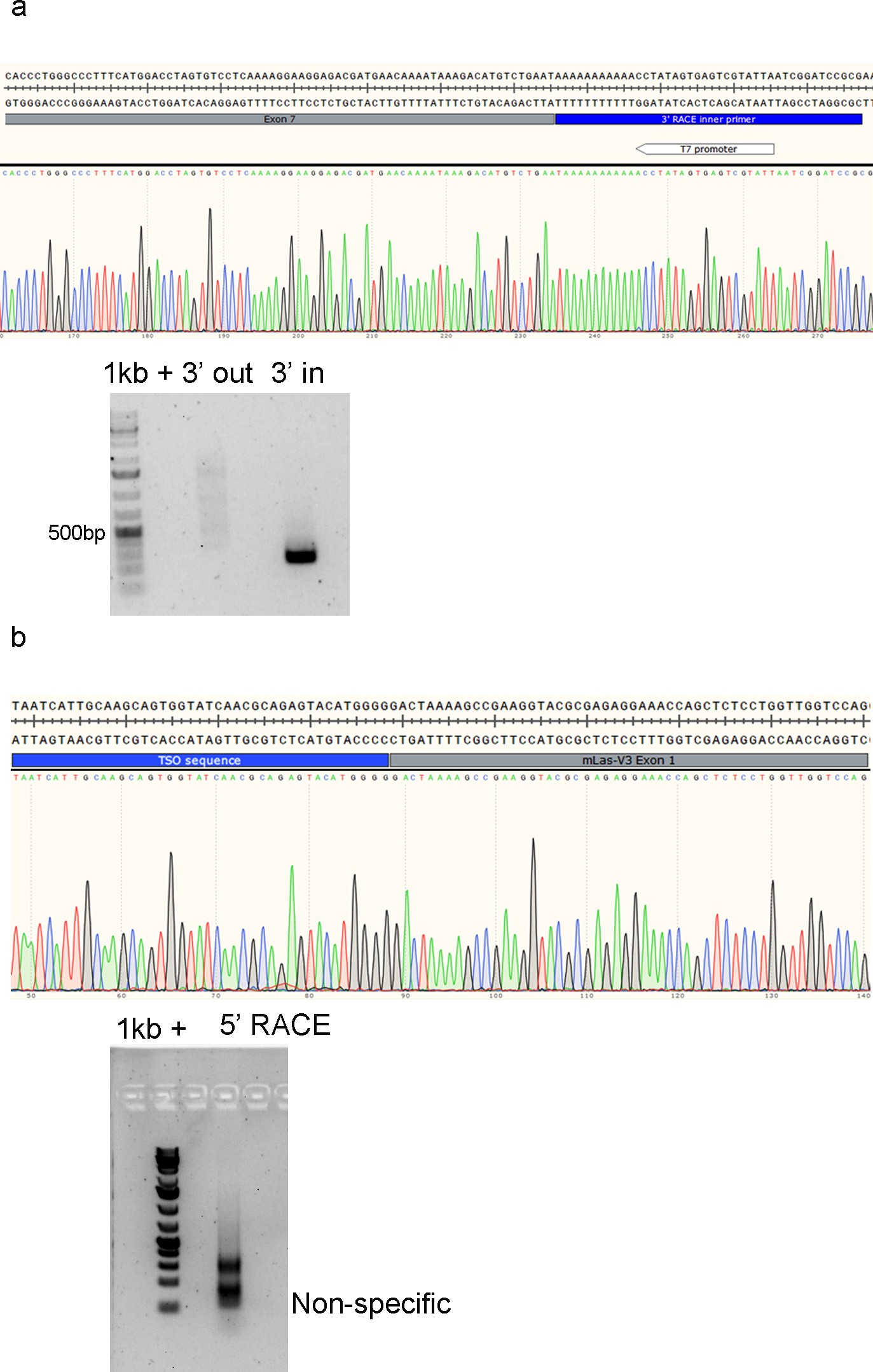


**Supplemental Figure S2. Localization of 5’-FAM labeled ASOs in cells undergoing differentiation.** Images of 5’-FAM labeled ASO and control treated cells taken at day 1, 2, 3, and 5 of differentiation at 10x magnification. At day 1, 5’-FAM ASOs are localized to the nucleus in all groups. However, after ASO treatment there is increased cell death leading to the accumulation of the 5’-FAM ASOs in apoptotic bodies. At day 5, some cells in the 5’-FAM ASO treated cells (see arrows) have accumulated lipid droplets. However, these cells do not have the 5’-FAM AOS signal suggesting that these cells were not successfully targeted by the ASOs.

**
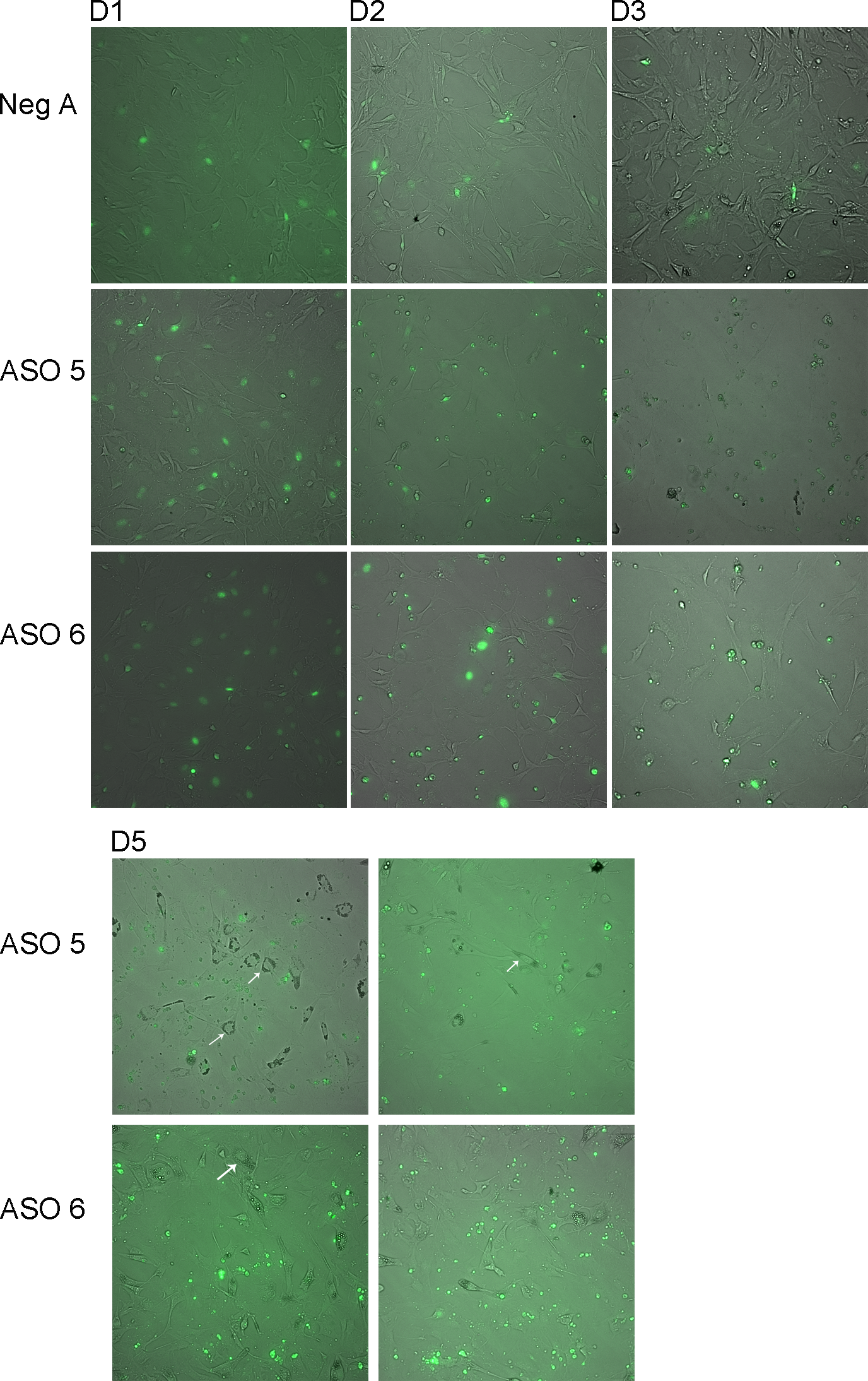
**
